# Mitotic MTH1 inhibitor TH1579 induces PD-L1 expression and inflammatory response through the cGAS-STING pathway

**DOI:** 10.1038/s41389-024-00518-1

**Published:** 2024-05-25

**Authors:** Jianyu Shen, Emilio Guillén Mancina, Shenyu Chen, Theodora Manolakou, Helge Gad, Ulrika Warpman Berglund, Kumar Sanjiv, Thomas Helleday

**Affiliations:** 1 Science for Life Laboratory, Department of Oncology-Pathology, Karolinska Institutet, Stockholm, Sweden; 2 Oxcia AB, Norrbackagatan 70C, 11334 Stockholm, Sweden; 3 Department of Oncology and Metabolism, Medical School, S10 2RX, Sheffield, UK

**Keywords:** Tumour immunology, Immunotherapy

## Abstract

The mitotic MTH1 inhibitor TH1579 is a dual inhibitor that inhibits mitosis and incorporation of oxidative DNA damage and leads to cancer-specific cell death. The response to immune checkpoint inhibitor (ICI) treatment is often augmented by DNA damaging agents through the cGAS-STING pathway. This study investigates whether TH1579 can improve the efficacy of immune checkpoint blockades through its immunomodulatory properties. Various human and murine cancer cell lines were treated with mitotic MTH1i TH1579, and the expression of PD-L1 and T-cell infiltration-related chemokines was analysed by flow cytometry and real-time qPCR. Syngeneic mouse models were established to examine the combined effect of TH1579 and PD-L1 blockade. In our investigation, we found that TH1579 upregulates PD-L1 expression at both the protein and mRNA levels in human cancer cell lines. However, in murine cell lines, the increase was less pronounced. An in vivo experiment in a syngeneic mouse melanoma model showed that TH1579 treatment significantly increased the efficacy of atezolizumab, an anti-PD-L1 antibody, compared to vehicle or atezolizumab monotherapy. Furthermore, TH1579 exhibited immune-modulatory properties, elevating cytokines such as IFN-β and chemokines including CCL5 and CXCL10, in a cGAS-STING pathway-dependent manner. In conclusion, TH1579 has the potential to improve ICI treatment by modulating immune checkpoint-related proteins and pathways.

## Introduction

Immune checkpoint inhibitors (ICIs) have generated a paradigm shift in cancer treatment, significantly prolonging overall survival compared to standard chemotherapy in certain cancers [1, 2]. Specifically, antibodies for blocking the interaction between programmed death-ligand 1 (PD-L1) and its receptor, PD-1, have been developed, enhancing treatment outcomes in non-small cell lung cancer (NSCLC), urothelial bladder cancer, and triple-negative breast cancer (TNBC) [3–5]. However, the response of ICIs varies among patients, with the level of PD-L1 expression in tumour cells and immunological hotness of tumours, being key determinants of therapeutic outcome. The FDA’s approval of atezolizumab for cancers with high PD-L1 expression illustrates this approach [6]. In NSCLC, ICI therapy benefits only 23–28% of patients, and predominantly those exhibiting high-level PD-L1 expression [7, 8]. Immunogenic hot tumours attract diverse T cells, which, upon treatment with PD-L1 blockades, activate them. This, in turn, effectively target tumour cells and results in tumour shrinkage [9, 10]. A limited number of cancers exhibit microsatellite instability (MSI) generating neo-antigens that improve ICI treatment classifying them as ‘hot’ tumours. However, many cancers are not MSI high and only a subset of patients obtain substantial benefits from ICIs [11, 12]. Therefore, transforming ‘cold’ tumours into ‘hot’ tumours is crucial for maximising the therapeutic efficacy of ICIs [9, 10].

Chemotherapeutic agents, radiation and targeted therapies can convert ‘cold’ tumours into ‘hot’ tumours not by introducing mutations as in the case for MSI high, but by modulating the tumour microenvironment, a process distinct from their direct mutagenic effects on cancer cells [12–14]. To argument the efficacy of ICIs, traditional chemotherapy drugs such as cisplatin, paclitaxel and carboplatin are co-administered with PD-1/PD-L1 blockades [15–18]. In a clinical trial study, a combination of atezolizumab and nab-paclitaxel treatment extended progression-free survival (PFS) by 2.5 months compared to placebo plus nab-paclitaxel in patients with PD-L1 positive TNBC though it was associated with an increase in serious adverse events (AEs) [19]. Meta analysis studies in NSCLC and hepatocellular carcinoma (HCC) have demonstrated that combination therapies improve overall survival (OS) and PFS, albeit with increased AEs, compared to monotherapy [15, 20–22]. Consequently, there is a critical need to develop new combination strategies that enhance the effectiveness of ICI therapies while mitigating AEs.

The human MutT homologue 1 (MTH1) protein is a nudix hydrolase that sanitises the cellular pool of nucleotides. It hydrolyses oxidised purine nucleoside triphosphates, such as 8-oxo-dGTP and 2-OH-dATP, into corresponding monophosphates, thereby preventing their erroneous incorporation into DNA and RNA [23, 24]. Recent studies have uncovered a role for MTH1 in in microtubule dynamics, revealing that its depletion leads to mitotic delays, lagging chromosomes and polynucleation [25, 26]. The mitotic MTH1 inhibitor TH1579 also impedes tubulin polymerisation in cancer cells. This dual inhibition confers broad anticancer activities to TH1579, which selectively induces cancer cell death through heightened ROS production, mitotic catastrophe and apoptosis [26–28].

Based on the link between oxidative DNA damage and PD-L1 [29, 30], we hypothesised that it might enhance the efficacy of anti-PD-1/PD-L1 therapy. This study aims to evaluate the impact of TH1579 on tumour PD-L1 expression and the antitumour immune response. Our findings indicate that TH1579 elevates PD-L1 expression and modulates the production of inflammatory cytokines and chemokines in cancer cells, suggesting a potential role in sensitising tumours to ICI treatment.

## Results

### Upregulation of PD-L1 by the mitotic MTH1 inhibitor TH1579

TH1579 induces cytotoxicity in a diverse array of cancer types, including both haematological malignancies and solid tumours [25, 26, 28, 31]. First, we determined the sensitivity of bladder cancer cell lines NTUB1 and UMUC3, as well as in the human lung cancer cell line A549 to TH1579 (Fig. S1A). All three cell lines were sensitive, and we established 0.5 or 1 μM as appropriate in vitro concentrations for analysing activity of TH1579 in human cancer cells.

Cisplatin (cis-diammine-dichloro-platinum II, CDDP) served as a positive control in our studies, owing to its well-documented ability to upregulate PD-L1 expression and exert immunomodulatory effects in cancer cells [32–34].

**Fig. 1:**
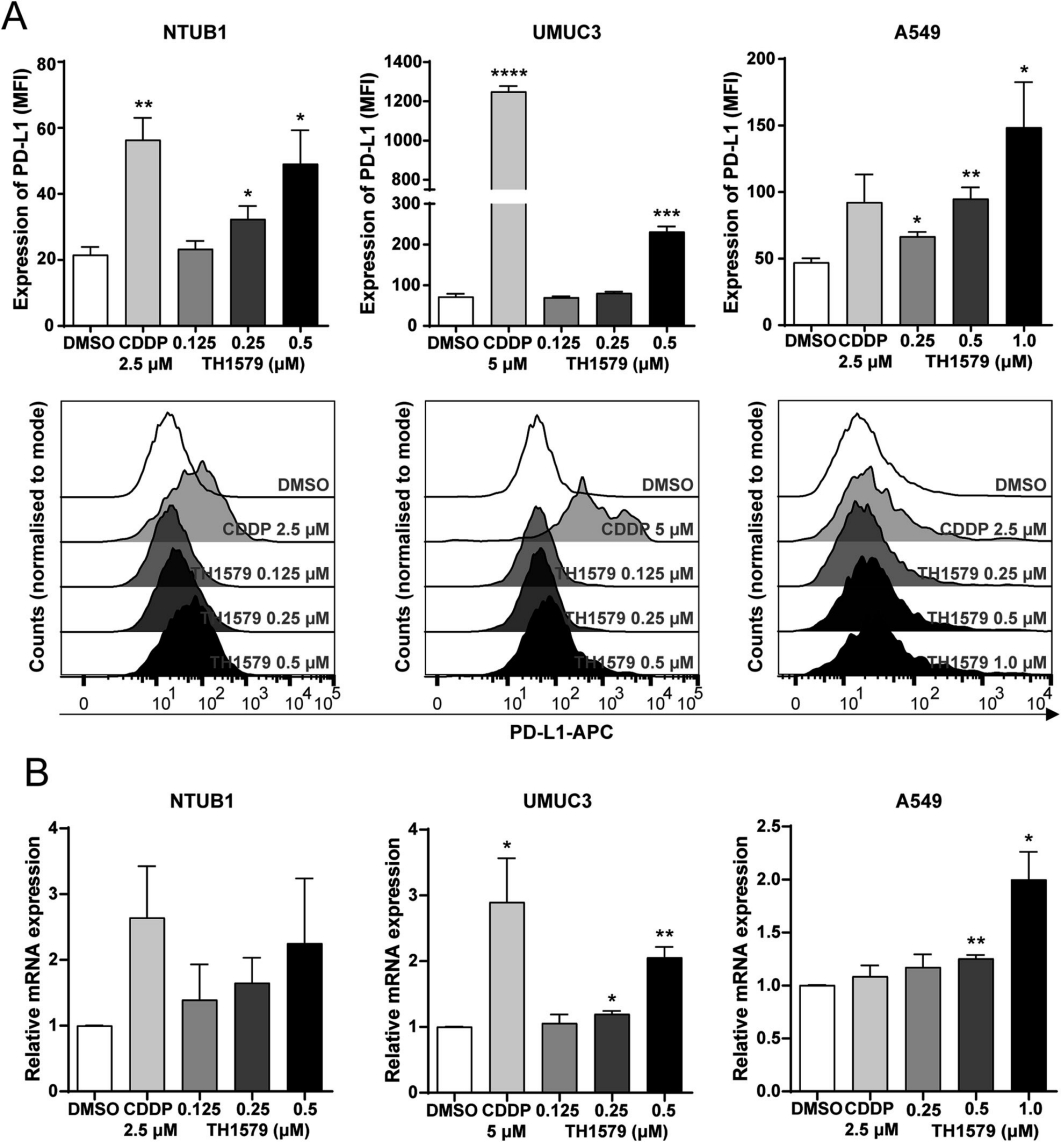
TH1579 elevates PD-L1 in different cancer cells at both expression and transcriptional levels. **A** Upper panel shows expression of PD-L1 in NTUB1, UMUC3, A549 cells was assessed by flow cytometry. Cells were treated with cisplatin or different concentration of TH1579 for 72 h. Median fluorescence intensity (MFI) was averaged from three independent experiments. The lower panel shows one of three independent experiments with comparable results (*n* = 5 for NTUB1). **B** Expression of *PDL1* in NTUB1, UMUC3, A549 cells was assessed by qPCR. Cells were treated with TH1579 or cisplatin for 48 h. The fold change in relative mRNA expression was averaged from three independent experiments. *p < 0.05, **p < 0.01, ***p < 0.001, ****p < 0.0001, Student’s *t* test.

To assess whether TH1579 induces PD-L1 expression in cancer cells, we conducted flow cytometry and real-time quantitative PCR (qPCR) across various human cancer cell lines. flow cytometry analysis revealed that a 72-h treatment with TH1579 significantly increased PD-L1 expression in NTUB1 and A549 cells, in a dose-dependent manner. However, such a dose-dependent increase was not observed in the UMUC3 cell line, which only show significant upregulation of PD-L1 at 0.5 µM TH1579 group (Fig. 1A). The mRNA levels, overall correlated with the increase of PD-L1 protein levels (Fig. 1B). The qPCR results in human colon carcinoma cell line HCT116 and uveal melanoma cell line MP41 showed the identical trend (Fig. S1B, C) These results indicate that TH1579 treatment broadly increases *PDL1* gene expression across a range of tumour types, potentially enhancing the responsiveness of these cells to anti-PD-L1 antibody therapy.

### Combination treatment with TH1579 and PD-L1 blockade in an in vivo murine model

**Fig. 2:**
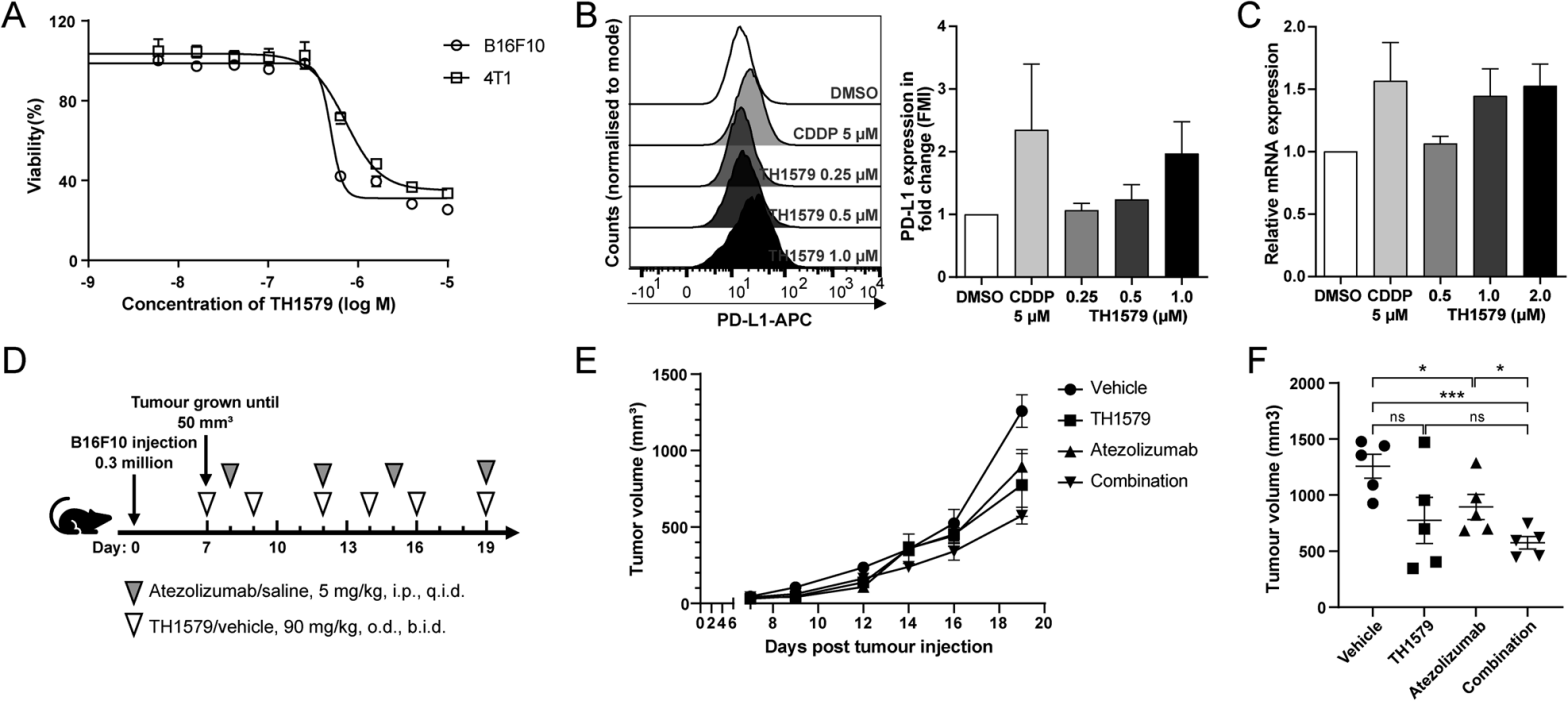
Combination treatment with TH1579 and PD-L1 blockade in in vivo murine model. **A** Dose-response curve of B16F10 and 4T1 cells treated with TH1579 for 72 h. Viability in different concentrations was averaged from four independent experiments. **B** Right: expression of PD-L1 in B16F10 cells was assessed by flow cytometry. Cells were treated with cisplatin or different concentration of TH1579 for 72 h. The fold change in median fluorescence intensity (MFI) was averaged from three independent experiments. Left: one of three independent experiments with comparable results. **C** Expression of *Pdl1* in B16F10 cells was assessed by qPCR. Cells were treated with TH1579 or cisplatin for 24 h. The fold change in relative mRNA expression was averaged from three independent experiments. **D**–**F** C57BL6/N mice were implanted with 0.3 × 10^6^ B16F10 cells and co-treated with TH1579 and Atezolizumab, *n* = 5. **D** A schema of the treatment plan. **E** Tumour growth curve. **F** Volume data of B16F10 tumours on day 19. *p < 0.05, ***p < 0.001, Student’s *t* test.

To evaluate the response of murine cancer cells to TH1579, we conducted cell viability assays on various mouse cancer cell lines, including the melanoma cell line B16F10, the colon cancer cell line CT-26, the breast cancer cell line 4T1, the kidney cancer cell line RenCa, and the lung cancer cell line LL2. Among them, B16F10 cell line exhibited the highest sensitivity, with an IC_50_ of ~0.50 ± 0.10 μM (Figs. 2A and S2A), roughly double of what were observed in human cancer cells. This differential sensitivity might be attributed to varying levels of MTH1 expression between human and murine cancer cell lines, and the poor inhibitory activity of TH1579 on the murine MTH1 protein. The basal expression of MTH1 in murine cancer cells, specifically B16F10 and 4T1, is relatively low (Fig. S2B). Conversely, the basal expression in NTUB1, UMUC3, and A549 is significantly elevated in comparison to murine cells (Fig. S2C). Given its relative sensitivity, the B16F10 cell line was selected for further investigation.

To determine if TH1579 similarly upregulates PD-L1 expression in murine cells as observed in human cancer cells, we assessed PD-L1 expression in B16F10 cell line using both qPCR and flow cytometry. Flow cytometry analysis revealed a <2-fold increase in PD-L1 expression in B16F10 cells treated with 0.5 μM TH1579 compared to those treated with DMSO (Fig. 2B). Notably, the extent of PD-L1 upregulation in B16F10 cells was lower than in human cancer cell lines, where it exceeded 2-fold (Fig. 1B). Additionally, TH1579 at 0.5 μM modestly upregulated the mRNA level of PD-L1 in B16F10 and 4T1 cell lines, although this increase was less substantial than that observed in NTUB1 and UMUC3 cell lines (Figs. 2C and S2D).

We assumed that the lack of significant PD-L1 upregulation in murine tumour cells after TH1579 treatment could be due to the inability of TH1579 to promote the incorporation of oxidised nucleotides such as 8-oxo-dGTP into the DNA. To investigate this possibility, we utilised a modified comet assay and found that treatment with TH1579 markedly increased 8-oxo-dGTP levels in all cell lines to an equal level (Figure S2E, S2F). These results suggest that there is no direct correlation between the impact of TH1579 on inducing DNA oxidative damage and PD-L1 expression.

Despite the less pronounced increase in PD-L1 expression by TH1579 in murine cancer cells as compared to human cells, we performed a proof-of-concept in vivo study using a B16F10 syngeneic allograft mouse model. Atezolizumab is a PD-L1 blockade and has been previously utilised in a syngeneic mouse model, either as a monotherapy or in combination with other inhibitors [35, 36]. From our previous studies, it is well documented that TH1579 significantly suppresses tumour growth in various human tumour xenograft in vivo models [26, 28]. In our proof-of-principal study, B16F10 allografted mice were treated with a regimen of vehicle, TH1579 90 mg/kg (b.i.d., 3 times a week), atezolizumab 5 mg/kg (q.i.d., twice a week), or a combination of both (Fig. 2D). Contrary to results in human xenografts [26, 28], TH1579 monotherapy did not significantly reduce tumour volume compared to the vehicle group, which is in line with poor activity of TH1579 on mouse cancers. Atezolizumab treatment statistically significant decreased the tumour volume (Fig. 2E, F). Furthermore, the combination of TH1579 and atezolizumab showed enhanced efficacy in tumour volume reduction and suppression compared to the vehicle treatment and atezolizumab monotherapy but not TH1579 treatment (Fig. 2E, F). This is anticipated as the TH1579 compound is 60-fold less potent inhibitor of the mouse versus human MTH1 protein [37]. However, this study suggests that TH1579 may potentiate the antitumour effect of atezolizumab. Further investigation in more appropriate in vivo models is necessary to substantiate this hypothesis.

### TH1579 treatment triggers a cytokine and chemokine response

**Fig. 3:**
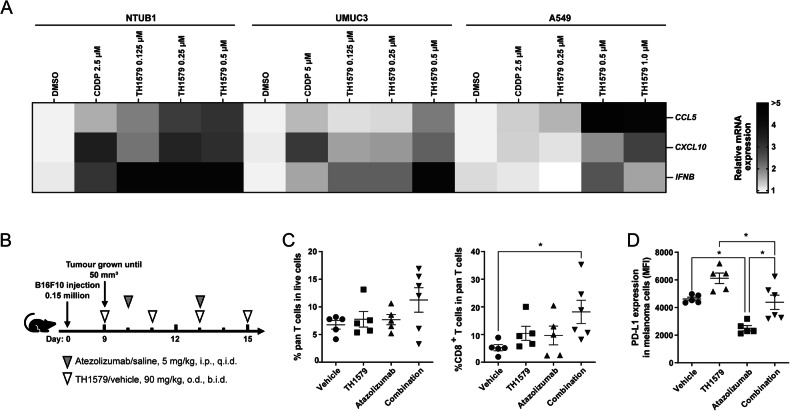
TH1579 elevates cytokines and chemokines which are related to CD8^+^ T cells infiltration. Transcriptional level expression of *CCL5, CXCL10, IFNB* in NTUB1, UMUC3 and A549. **A** The heatmap and clustering of 3 target genes based on their expressions in 3 tumour cell lines. Cells were treated with different concentrations of TH1579 or cisplatin for 48 h. The fold change in relative mRNA expression was averaged from two independent experiments. *p < 0.05, **p < 0.01, ***p < 0.001, ****p < 0.0001, Student’s *t* test. **B**–**D** C57BL6/N mice were implanted with 0.15 × 10^6^ B16F10 cells and co-treated with TH1579 and Atezolizumab, *n* = 5 or 6. **B** A schema of the treatment plan. **C** Quantification of flow cytometry of CD3^+^ T cells in live cell, CD8^+^ T cells in CD3^+^ T cells. **D** MFI of PD-L1 in melanoma B16F10 cells (CD45^-^, gp-100^+^). *p < 0.05, One way ANOVA.

Chemotherapy drugs such as cisplatin and mitotic poisons such as paclitaxel and vincristine are known to modulate the tumour microenvironment [18, 30]. Due to a similar mode of action, we also hypothesised that the mitotic MTH1 inhibitor TH1579 may activate antitumour immunity in the cancer microenvironment. To validate this hypothesis, we treated various cancer cell lines with TH1579 and observed a significant increase in the mRNA expression of the chemokines *CCL5* and *CXCL10* in NTUB1 and UMUC3 cells at 0.5 μM, and in A549 cells at 1 μM (Figs. 3A and S3A–C). Additionally, we detected elevated mRNA expression of type I interferon (*IFNB*) following TH1579 treatment in NTUB1 and UMUC3 cells across a concentration range of 0.125– 0.5 μM (Fig. 3A). However, in A549 cells, TH1579 treatment did not alter the transcriptional level of *IFNB*, likely due to the low basal expression in these cells, rendering accurate CT value determination challenging (Fig. S3C). In murine cancer cell lines B16F10 and 4T1, TH1579 treatment at 1 μM for 24 h resulted in increased transcription of *Ccl5* and *Cxcl10* (Fig. S3D, E).

In the tumour microenvironment, CCL5 plays a crucial role in recruiting dendritic cells that predominantly present antigens to CD8^+^ T cells, and CXCL10 enhances the infiltration of CD8^+^ T cells into tumours [38–40]. To ascertain whether TH1579 can enhance the infiltration of CD8^+^ T cells within the tumour microenvironment, B16F10 allografted mice were subjected to a treatment regimen comprising either a vehicle, TH1579 at a dosage of 90 mg/kg (b.i.d., 3 times a week), atezolizumab at a dosage of 5 mg/kg (q.i.d., twice a week), or a combination of both (Fig. 3B). The combination treatment of TH1579 and Atezolizumab resulted in a significantly higher infiltration of CD8^+^ T cells compared to the vehicle group. However, this difference was not statistically significant when compared to either the TH1579 treatment or the Atezolizumab treatment (Fig. 3C).

In addition, we evaluated the expression of PD-L1 in tumour cells from the same in vivo study. Interestingly, the group treated with the combination did not exhibit the highest PD-L1 expression. However, when comparing the vehicle group to the TH1579 treatment group and the Atezolizumab treatment group to the combination group, the PD-L1 expression was found to be higher in groups where TH1579 was present (Fig. 3D). Conversely, the PD-L1 expression in groups where Atezolizumab was present (Atezolizumab treatment group and combination group) was lower than in the corresponding groups where Atezolizumab was absent (vehicle group and TH1579 treatment group, respectively). This could potentially be attributed to the fact that Atezolizumab primarily targets PD-L1^high^ tumour cells, and we analysed PD-L1 expression in live cells in this study.

Therefore, our findings suggest that TH1579 can module the tumour microenvironment, potentially aiding in the recruitment of CD8^+^ T cells to the tumour site, which could underline the enhanced antitumour efficacy observed with the drug combination.

### PD-L1 and cytokine response by TH1579 is cGAS-STING dependent

**Fig. 4:**
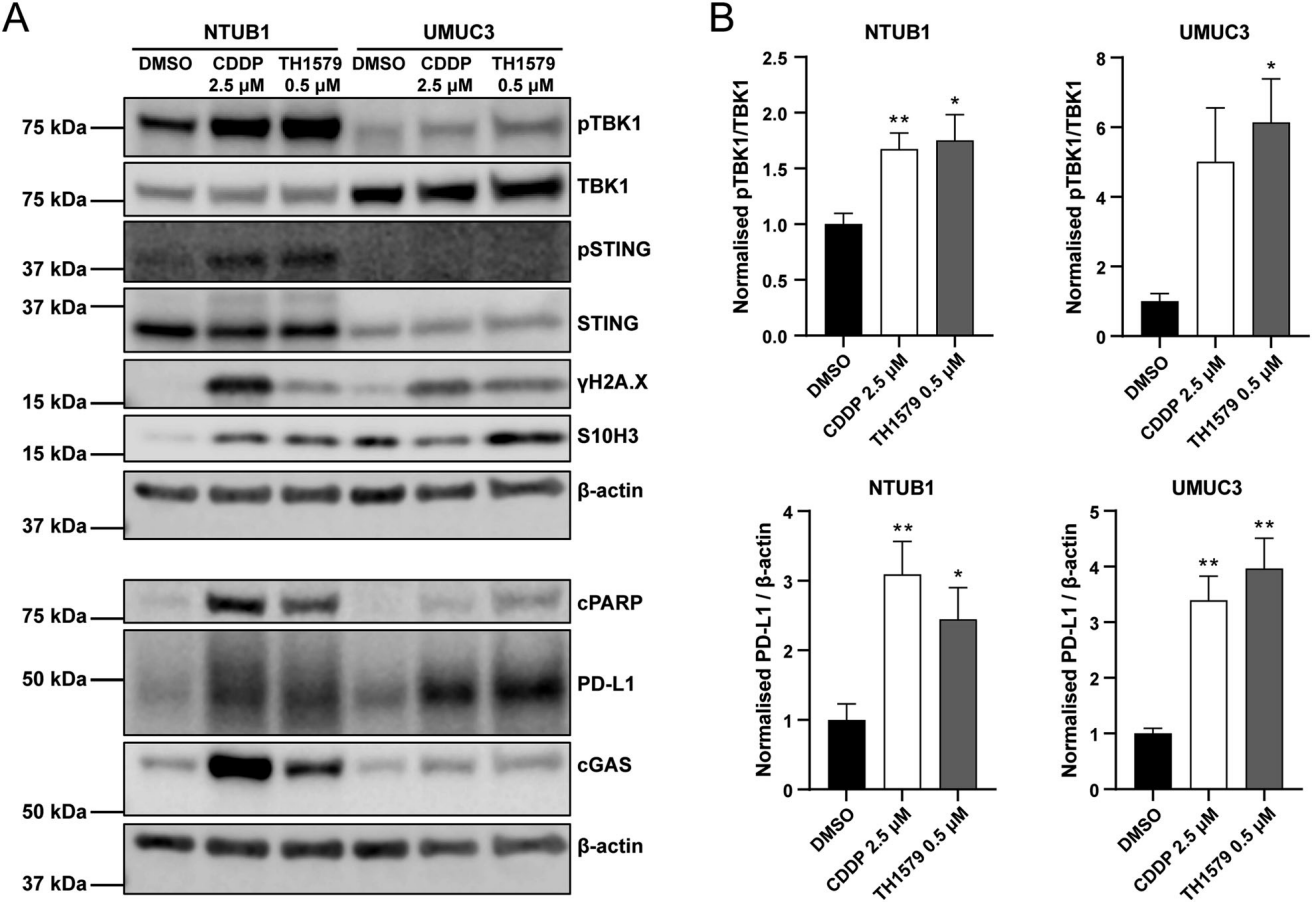
TH1579 activates cGAS-STING pathway. NTUB1 and UMUC3 cells were cultured in 2.5 µM cisplatin or 0.5 µM TH1579 for 72 h and lysates prepared for western blot analysis with indicated antibodies. **A** Representative blot. **B** Quantification of bands. The fold change in protein expression was normalised by β-actin and averaged from three independent experiments (*n* = 5 for NTUB1). *p < 0.05, **p < 0.01, Student’s *t* test.

The presence of mitotic DNA in the cytoplasm can trigger activation of the cGAS-STING pathway, a critical innate immune response within the tumour microenvironment. To investigate whether TH1579 actives this pathway, we treated NTUB1 and UMUC3 cells with 0.5 μM TH1579 for 72 h and examined the activation of related proteins. The time point for treatment was decided according to the time-course experiment in NTUB1 cells (Fig. S4). Our results demonstrate that TH1579 increases TBK1 phosphorylation in both cell lines with a significant elevation, suggesting that TH1579 can indeed activate the cGAS-STING pathway (Fig. 4).

**Fig. 5:**
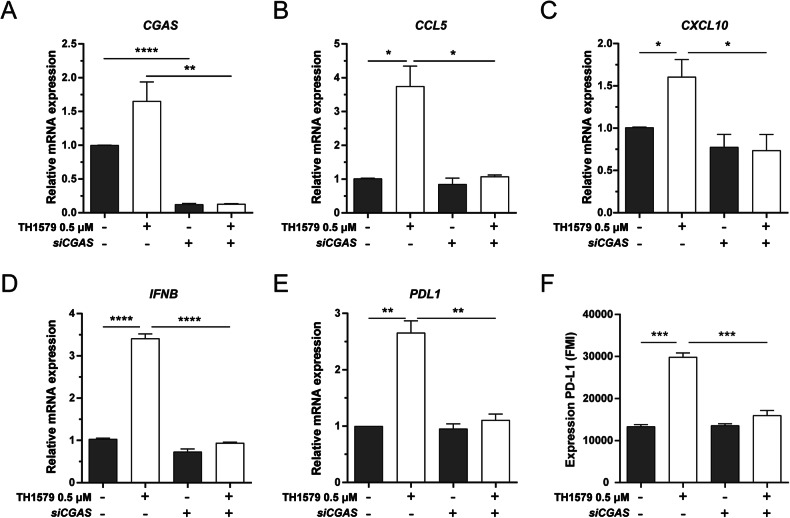
PD-L1 and cytokine response by TH1579 is cGAS-STING dependent. UMUC3 cells were transferred with si*CGAS* for 24 h followed by DMSO or 0.5 µM TH1579 treatment for 48 h. **A** *CGAS*, **B** *CCL5*, **C** *CXCL10*, **D** *IFNB* and **E** *PDL1* were detected at mRNA level measured by qPCR. The fold change in relative mRNA expression was averaged from three independent experiments. **F** NTUB1 cells were transferred with si*CGAS* for 24 h followed by DMSO or 0.5 µM TH1579 treatment for 72 h then measured PD-L1 expression by flow cytometry. The fold change in median fluorescence intensity (MFI) was averaged from three independent experiments. *p < 0.05, ***p < 0.001, Student’s *t* test.

IFN-β is primarily induced through STING-dependent signalling [41, 42]. CCL5 and CXCL10 are also associated with STING/TBK1/IRF3-dependent pathway [43–45]. Furthermore, STING activation has been shown to upregulate PD-L1 expression [34, 46]. In light of these associations, we further assessed whether TH1579 could enhance the expression of PD-L1, CCL5, CXCL10 and IFN-β through the cGAS-STING pathway. Upon cGAS depletion in UMUC3 cells (confirmed by qPCR; Fig. 5A), followed by TH1579 treatment, we observed a substantial reduction in the mRNA levels of *CCL5*, *CXCL10*, *IFNB* and *PDL1* (Fig. 5B–E). This was also reflected in the protein expression level of PD-L1 (Fig. 5F). However, in NTUB1 cells, cGAS knockdown significantly diminished *CCL5*, *CXCL10*, *IFNB* and *PDL1* (Fig. S5B–E), but this was not mirrored at the protein level of PD-L1 (Fig. S5F). In conclusion, our data indicates that TH1579 has the potential to enhance the expression of PD-L1, CCL5, CXCL10 and IFN-β by activating the cGAS-STING pathway.

## Discussion

Here, we demonstrate that TH1579, a mitotic MTH1 inhibitor, has a potential to enhance the efficacy of ICIs by upregulating PD-L1, CCL5, CXCL10 and IFN-β according to in vitro experiments (Figs. 1 and 3). Previous studies have illustrated that increased PD-L1 expression can augment the effectiveness of immunotherapy across various cancer types [47, 48]. Clinically, it has been observed that NSCLC patients with high PD-L1 expression (defined as over 1–50% in different clinical trials) exhibit longer PFS compared to those with low expression [49, 50]. In the tumour microenvironment, certain cytokines and chemokines are involved in the recruitment of CD8^+^ T cells. For example, in small cell lung cancer (SCLC), a WEE1 inhibitor induced DNA damage in cancer cells, increasing type I interferons, CCL5 and CXCL10 via activation of the cGAS-STING pathway, enhancing the response to PD-L1 blockade. This was accompanied by an increased presence of CD8^+^ T cells at the tumour site [51]. Similarly, research involving PARP inhibitors like Olaparib has shown activation of the cGAS-STING pathway, resulting in increased CD8^+^ T cell infiltration and enhanced efficacy of anti-PD-1/PD-L1 treatments [45, 52]. These studies lend support to the proposition that TH1579 could be an effective candidate for combination with immunotherapy.

While TH1579 demonstrates potential for combination with PD-L1 blockade in human cancer cells, the mouse model did not exhibit a robust synergistic effect. TH1579 monotherapy at 90 mg/kg did not reduce tumour size significantly in vivo, contrasting with its promising efficacy observed in human HL-60, THP-1 and SW480 xenograft models at the same dose [26, 28]. A possible explanation for the limited response in the syngeneic mouse model could be the differential affinity of TH1579 to inhibit the murine MTH1 protein as compared to human MTH1 [37]. In vitro assays including dose response curves and PD-L1 expression levels in different murine cell lines also indicated that some murine cancer cells might not respond TH1579 as sensitively as human cancer cells (Figs. 2A–C and S2A–D). A recent study combining TH1579 with anti-PD-L1 immunotherapy in mesothelioma syngeneic models revealed significant responses. The AE17 model showed limited response to TH1579 monotherapy compared to the AB1 model, whereas AB1 model had no effect of combined treatment [53]. In vivo models can reveal how the immune system responds to different compounds and antibodies, which is a key aspect in immunotherapy research. In general, the syngeneic mouse model is widely used since its ease of establishment and its capacity to avoid immune responses from xenogeneic tumours [54]. Additionally, genetically engineered murine cancer cell lines are used to replicate human genotypes in some studies [55]. Moreover, humanised mouse models, which are immunodeficient mice engrafted with human peripheral blood mononuclear cells (PBMCs) or hematopoietic stem cells (HSCs), have been used for immunotherapy [56, 57]. Given the potential for a more pronounced response, future studies will explore effects of TH1579 in these more sophisticated models.

## Materials and methods

### Cell culture

UMUC3 (human bladder carcinoma) and A549 (human lung adenocarcinoma), HCT116 (human colon carcinoma), MP41 (human uveal melanoma) were purchased from ATCC. B16F10 (mouse melanoma), 4T1 (mouse malignant neoplasms of mammary gland), LL2 (mouse Lewis lung carcinoma), Renca (mouse kidney carcinoma) and CT26 (mouse colon adenocarcinoma) were gifts from Prof. Miguel López Lázaro, Department of Pharmacology, University of Seville, Spain. NTUB1 (human bladder carcinoma) was a gift from Prof. Te-Chang Lee, IBMS, Academia Sinica, Taipei Tiwan. A549 and B16F10 cells were cultured in DMEM with GlutaMax. NTUB1, 4T1, LL2, Renca, CT26 and MP41 cells were cultured in RPMI1640 with GlutaMax, UMUC3 cells were cultured in Minimum Essential Medium with GlutaMax and HCT116 cells were cultured in McCoy’s 5 A Medium with GlutaMax. Media were supplemented with 10% heat-inactivated FBS, 100 U/ml penicillin and 100 μg/ml streptomycin. All cells were maintained at 37 °C, 5% CO_2_ and a humid incubator.

### Compounds and blockades

TH1579 was prepared according to published methods (WO2015187088). Cisplatin was purchased from Sigma-Aldrich. Atezolizumab (Tecentriq, 1200 mg) was from Roche.

### Cell viability

TH1579 was dissolved in dimethyl sulfoxide (DMSO) at 10 mM and dispensed to final concentration using D300e digital dispenser (Tecan). Cells were seeded in the described complete media, and plates were incubated for 96 h. Cell viability was determined by adding 10 μg/mL resazurin (Sigma Aldrich) and measured after 4–6 h. Fluorescence at 595 nm was measured by Hidex Sense reader. Half-inhibition concentration (IC_50_) was calculated in GraphPad Prism v.9.4.1

### Mice and treatment

All animal experiments were approved and conducted as per the European directive, ethical guideline, and regulations of the Institutional Review Committee, that is, Regional Animal Ethical Committee Stockholm (approval Dnr: 5718-2019). C57BL6/N female mice were purchased from Charles River. All mice (6–8 weeks old) were housed in 3–5 mice / cage with a 12-h light cycle. Temperature and humidity set according to laboratory animal guidelines and regulation.

0.3 million (or 0.15 million) B16F10 cells were injected subcutaneously at the right flank to generate a syngeneic model. Animals were randomised into treatment groups when tumours reached 50 mm^3^. Animals were euthanized when human endpoints were reached.

TH1579 (90 mg/kg, twice daily, per oral, p.o.) was formulated in a vehicle solution of 22.5% Hydroxypropyl-β-cyclodextrin with sterile water. Atezolizumab (diluted to 0.5 mg/mL) was formulated in saline.

### Tumour dissociation and flow cytometry

Mice were sacrificed at the indicated days. Tumours were extracted, finely minced and digested with the MACS Miltenyi Tumor Dissociation Kit (Miltenyi Biotec) according to the manufacturer’s instructions. Dissociated tumour cells were washed with RPMI-1640 medium and lysed with ACK Lysing Buffer (Gibco). Cells were resuspended in staining buffer (DPBS with 5% FBS and 2 mM EDTA). LIVE/DEAD™ Fixable Aqua Dead Cell Stain Kit (Invitrogen) was applied to cells in combination with Rat anti-mouse CD16/CD32 Fc Block (BD Biosciences, #553142) for 10 min at room temperature, prior to incubation with antibodies for 45 min at 4 °C. For immune cell staining penal, cells were fixed with 2% PFA for 30 min and washed, resuspended in staining buffer. For tumour cell penal, as gp-100 is an intracellular marker, cells were fixed and permeabilized with Foxp3 Transcription factor staining buffer kit (Thermo Fisher) according to the manufacturer’s instructions, followed by incubation with antibodies for 60 min at 4 °C. Compensation was performed using UltraComp eBeads™ Plus Compensation Beads (Invitrogen) incubated with antibodies and ArC™ Amine Reactive Compensation Bead Kit (Invitrogen) incubated with LIVE/DEAD staining. Signal threshold definition was defined using all-stain, unstained, and FMO controls. Gating strategies are provided in Supplementary Fig. S6. Samples were analysed on NovoCyte Flow Cytometer (Aglient) and data was analysed by FlowJo v.10.8.1.

Following antibodies were used: BV786 Rat Anti-Mouse CD45 (BD Bioscience, clone 30-F11, 1:100), BV711 Hamster Anti-Mouse CD3e (BD Bioscience, clone 145-2C11, 1:100), Pacific Blue™ Rat Anti-Mouse CD8a (BD Bioscience, clone 53-6.7, 1:100), PE Anti-Melanoma gp100 (Abcam, ab246731, 1:5000).

### Western blot

Cell pellets were incubated on ice in lysis buffer (50 mM Tris-HCl pH 8.0, 150 mM NaCl, 1 mM EDTA, 1% NP40, 0.1% SDS, 1x protease inhibitor cocktail (Sigma-Aldrich), and 1x Halts phosphatase inhibitor cocktail (Thermo Fisher Scientific)) and sonicated. Lysate was centrifuged at 13,000 rpm, 20 min to collect supernatant. Protein concentration was determined by BCA (Bicinchoninic Acid) Protein Assay (Thermo Fisher Scientific). Samples were prepared in NuPAGE™ LDS sample buffer (Invitrogen) with NuPAGE™ Sample Reducing Agent (Invitrogen). Samples were denatured at 70 °C for 10 min. Samples were loaded on 4–15% SDS-PAGE gel (Criterion™ TGX™ Precast Midi Protein Gel, Bio-Rad) and the proteins were transferred to nitrocellulose membranes using the Trans-Blot Turbo instrument (Bio-Rad) according to the standard protocol. Membranes were stained with Ponceau S and blocked in 5% milk powder or 1% BSA in tris-buffered saline with Tween and then probed with primary antibodies over night at 4 °C. Secondary antibodies were probed for 2 h at room temperature. Images of blots were obtained using the LI-COR Odyssey Fc Imaging system (LI-COR) and analysed by ImageStudioLite v.5.2 (LI-COR).

### Antibodies

Following antibodies were used: mouse anti beta-Actin (Abcam, ab6276, 1:1000), mouse anti-H2A.X phospho S139 (Millipore, 05-636, 1:1000), mouse anti-Histone H3 phospho-S10 (H3-pS10; Abcam, ab5176, 1:1000), rabbit anti-cleaved PARP (Cell Signaling, #9541, 1:1000), rabbit anti-PDL1 (Cell Signaling, #13684, 1:1000), rabbit anti-pTBK1 (Cell Signaling, #5483, 1:500), rabbit anti-TBK1 (Cell Signaling, #3504, 1:1000), rabbit anti-pSTING (Cell Signaling, #50907, 1:500), rabbit anti-STING (Cell Signaling, #13647, 1:1000), rabbit anti-cGAS (Cell Signaling, #15102, 1:1000), rabbit anti-MTH1 (Novus Biologicals, NB100-109, 1:1000). Secondary antibodies were: Peroxidase AffiniPure Donkey Anti-Rabbit IgG (Jackson ImmunoResearch, 711-035-152, 1:5000), Peroxidase AffiniPure Donkey Anti-Mouse IgG (Jackson ImmunoResearch, 715-035-150, 1:5000), IRDye 680RD Goat Anti-Mouse IgG (LI-COR, 926-68072, 1:5000) and IRDye 800CW Donkey Anti-Rabbit IgG (LI-COR, 926-32213, 1:5000).

### Transfection of siRNA

To established cGAS knock down cells, *CGAS* siNRA (SMARTpool) was purchased from Horizon Discovery. 20 nM (for NTUB1) or 10 nM (for UMUC3) of siRNA was transfected by using INTERFERin transfection reagent according to manufacturer’s instruction. The same concentration of All-Stars negative control (Qiagen) was used as non-targeting control. After transfection, cells stilled at least 48 h (for NTUB1) or 24 h (for UMUC3) and then add compounds for treatment.

### PD-L1 expression analysis by flow cytometry

Cells were cultured with different compounds. After treatment, cells were collected, washed and resuspended in DPBS with 5% FBS and 2 mM EDTA. Cells were stained with APC conjugated rat anti-mouse CD274 (BD Biosciences, clone MIH5, 1:100) for murine cells or APC conjugated mouse anti-human CD274 (BioLegend, clone 29E.2A3, 1:40) for human cells, or isotype controls including APC Rat IgG2a, λ Isotype Control for anti-mouse CD274 (BD Biosciences, clone B39-4, 1:100), APC Mouse IgG2b, κ Isotype Ctrl (Biolegend, clone MPC-11, 1:40) in dark. Cells were washed and resuspended in DPBS with 5% FBS and 2 mM EDTA. Representative gating strategies are provided in Supplementary Fig. S1D. Samples were analysed on Navios flow cytometer (Beckman Coulter) and data was analysed by FlowJo v.10.8.1.

### RT-qPCR

Cells were collected by scraping in TRI Reagent (Zymo Research). Total RNA was prepared with the Direct-zol RNA miniprep kit (Zymo Research) and cDNA was prepared with QuantiTect Reverse Transcriptase kit (Qiagen) according to manufacturer’s instructions. Each reaction contained 40 ng of cDNA, 1 µM forward and reverse primers and 1x iTaq universal SYBR green supermix (Bio-Rad). The qPCR reactions were performed in a Rotor-Gene Q instrument (Qiagen). Each qPCR reaction was made in triplicates and expression of target genes were normalised to the control geneactin beta.

Following primers for human were used:

*PDL1*_Forward: 5′-CCTCCAAATGAAAGGACTCAC-3′

*PDL1*_Reverse: 5′-TTTTCACATCCATCATTCTCCC-3′

*CCL5*_Forward: 5′-TGCCACTGGTGTAGAAATACTC-3′

*CCL5*_Reverse: 5′-GCTGTCATCCTCATTGCTACT-3′

*CXCL10*_Forward: 5′-GACATATTCTGAGCCTACAGCA-3′

*CXCL10*_Reverse: 5′-CAGTTCTAGAGAGAGGTACTCCT-3′

*IFNB*_Forward: 5′-AACTTGCTTGGATTCCTACAAAG-3′

*IFNB*_Reverse: 5′-TATTCAAGCCTCCCATTCAATTG-3′

*ACTB*_Forward: 5′-CATTGCTGACAGGATGCAGAAGG-3′

*ACTB*_Reverse: 5′-TGCTGGAAGGTGGACAGTGAGG-3′

Following primers for mouse were used:

*Pdl1*_Forward: 5′-CCACATTTCTCCACATCTAGCA-3′

*Pdl1*_Reverse: 5′-TCCATCCTGTTGTTCCTCATTG-3′

*Ccl5*_Forward: 5′-CCTCTATCCTAGCTCATCTCCA-3′

*Ccl5*_Reverse: 5′-GCTCCAATCTTGCAGTCGT-3′

*Cxcl10*_Forward: 5′-ATTTTCTGCCTCATCCTGCT-3′

*Cxcl10*_Reverse: 5′-TGATTTCAAGCTTCCCTATGGC-3′

*Ifnb*_Forward: 5′-CCAGCTCCAAGAAAGGACGA-3′

*Ifnb*_Reverse: 5′-CGCCCTGTAGGTGAGGTTGAT-3′

*Actb*_Forward: 5′-CATTGCTGACAGGATGCAGAAGG-3′

Actb_Reverse: 5′-TGCTGGAAGGTGGACAGTGAGG-3′

### Modified comet assay

Cells were seeded in 6-well plates at a density of 300,000–400,000 cells/well and the next day treated with TH1579 or DMSO for 24 h. Cells were harvested and washed once with DPBS and finally resuspended in DPBS at a concentration of 1 million/mL. 100 μL of cell suspension was mixed with 500 μL 1.2% low melting point agarose at 37 °C and the mixture were added to agarose coated slides and a coverslip was added on top. The slides were lysed overnight at 4 °C in Lysis buffer (2.5 M NaCl, 100 mM EDTA, 10 mM Tris, 10% DMSO, 1% Triton X100). Slides were washed three times in enzyme buffer (40 mM HEPES, 0.1 M KCl, 0.5 mM EDTA, 0.2 g/L BSA, pH 8.0) and treated with hOGG1 enzyme (2 µg/mL) or buffer alone for 45 min at 37 °C. Slides were transferred to alkaline electrophoresis buffer (300 mM NaOH, 10 mM EDTA) for 20 min and electrophoresis was performed at 25 V, 300 mA for 30 min at 4 °C. Slides were washed in Neutralization buffer (400 mM Tris, pH 7.5) for 45 min. DNA was stained with SYBR gold dye (Thermo Fisher Scientific), and comets were imaged and quantified with Comet Assay IV software.

### Quantification and statistical analysis

All data were plotted and statistical analysis was carried out in GraphPad Prism v.9.4.1. Data were plotted as means ± standard error mean (SEM).

### Supplementary information

Supplemetary figures and legends

## Data Availability

The authors declare that the data supporting the findings of this study are available within the paper and its Supplementary Information files. Should any raw data files be needed in another format they are available from the corresponding author upon reasonable request.
