# Supplementary material for: Mitotic MTH1 inhibitor TH1579 induces PD-L1 expression and inflammatory response through the cGAS-STING pathway
Source: Oncogenesis. 2024 May 25;13(1):17. doi: 10.1038/s41389-024-00518-1 (PMC11127983; doi:10.1038/s41389-024-00518-1)
Supplement: Supplementary file 1 — Supplemetary figures and legends [file 41389_2024_518_MOESM1_ESM.pdf]

FIGURE LEGENDS FOR SUPPLEMENTARY FIGURES

Supplemental figure 1. TH1579 elevates the expression of PD-L1 at mRNA level in human colon cancer and uveal melanoma cells.

A. Dose-response curve of NTUB1, UMUC3 and A549 cells treated with TH1579 for 72 hours. Viability in different concentrations was averaged from four independent experiments. Expression of *PDL1* in **B.** HCT116 and **C.** MP41 cells was assessed by qPCR. Cells were treated with different concentrations of TH1579 or cisplatin for 48 hours. The fold change in relative mRNA expression was averaged from one experiment for HCT116 and two independent experiments for MP41. *$p < 0.05$, **$p < 0.01$, Student's t test. **D.** Representative flow cytometry gating strategy for the identification of tumour cells. Isolated tumour cells were gated on size, singularity, viability. Median fluorescence intensity (MFI) of APC was analysed in live cells.

Supplemental figure 2. PD-L1 expression at transcriptional level by treated with TH1579.

A. Dose-response curve of CT26, LL/2, RenCa, 4T1 and B16F10 cells treated with different concentrations of TH1579 for 72 hours. Viability in different concentrations was averaged from four independent experiments. **B.** Expression of *Pdl1* in 4T1 cells was assessed by qPCR. Cells were treated with different concentrations of TH1579 or cisplatin for 24 hours. The fold change in relative mRNA expression was averaged from two independent experiments. *$p < 0.05$, Student's t test. **C.** Representative blot of B16F10 and 4T1 cells. Cells were cultured in DMSO or 1.0 µM TH1579 for 72 hours and lysates prepared for Western Blot analysis with indicated antibodies, n=2. **D.** Representative blot of NTUB1, UMUC3, A549, B16F10, 4T1 cells. Cells were cultured in media and lysates prepared for Western Blot analysis with indicated antibodies, n=2. **E** and **F.** NTUB1, UMUC3, A549,

B16F10 and 4T1 cells were treated with TH1579 for 24 hours. Genomic levels of 8-oxo-dG were analysed using modified comet assay. **E.** Representative images of NTUB1. **F.** Quantification of Tail moment, at least 200 cells in two independent experiments were analysed. ****p < 0.0001, One-Way ANOVA.

Supplemental figure 3. Expression of cytokines and chemokines at transcriptional level in murine cells.

A-C. Histograms of individual gene expression fold changes in **B.** NTUB1, **C.** UMUC3 and **D.** A549 from Figure 3A. Transcriptional level expression of *Ccl5*, *Cxcl10*, and *Ifnb* in **D.** B16F10 and **E.** 4T1 cells was assessed by qPCR. Cells were treated with different concentrations of TH1579 or cisplatin for 24 hours. The fold change in relative mRNA expression was averaged from two independent experiments. *p < 0.05, **p < 0.01, ****p < 0.0001, Student's t test.

Supplemental figure 4. TH1579 activates cGAS-STING pathway after 48-hour treatment

NTUB1 cells were cultured in 0.5 µM TH1579 for 0, 24, 48 or 72 hours and lysates prepared for Western Blot analysis with indicated antibodies.

Supplemental figure 5. PD-L1 and cytokine response by TH1579 is cGAS-STING dependent in NTUB1 cells

NTUB1 cells were transferred with si*CGAS* for 48 hours followed by DMSO or 0.5 µM TH1579 treatment for 48 hours. **A.** *CGAS*, **B.** *CCL5*, **C.** *CXCL10*, **D.** *IFNB* and **E.** *PDL1* were detected at mRNA level measured by qPCR. The fold change in relative mRNA expression was averaged from three independent experiments. **F.** NTUB1 cells were transferred with si*CGAS* for 48 hours followed by DMSO or 0.5 µM TH1579 treatment for 72 hours then measured PD-L1 expression by flow cytometry. The fold change in median fluorescence intensity (MFI)

was averaged from three independent experiments. *p < 0.05, ***p < 0.001, ****p < 0.0001, Student’s t test.

Supplemental figure 6. Gating strategy for *in vivo* study (relates to Figure 3)

A. Representative flow cytometry gating strategy for the identification of $CD8^+$ T cell populations in tumours from B16F10 model. Isolated $CD8^+$ T cells were first gated on size, singularity, viability and positive expression of CD45 and CD3. **B.** Representative flow cytometry gating strategy for the identification of B16F10 cell populations in tumours from B16F10 model. Isolated tumour cells were first gated on size, singularity, viability, negative expression of CD45 and positive expression of gp-100.

Supplemental figure 7. Isotype control validation and basal of PD-L1 in different cell lines (relates to Figure 1)

Representative histograms for PD-L1 expression in NTUB1, UMUC3 and A549 with different treatments. Cells were seeded 200,000 in each well and treated with indicated compounds for 72 hours. After that, cells were collected and stained with PD-L1 antibody and isotype control with 1:40 dilution. Cells were gated according to the gating strategy in **Figure S1D**. N=2, for cisplatin group, n=1

Figure S1

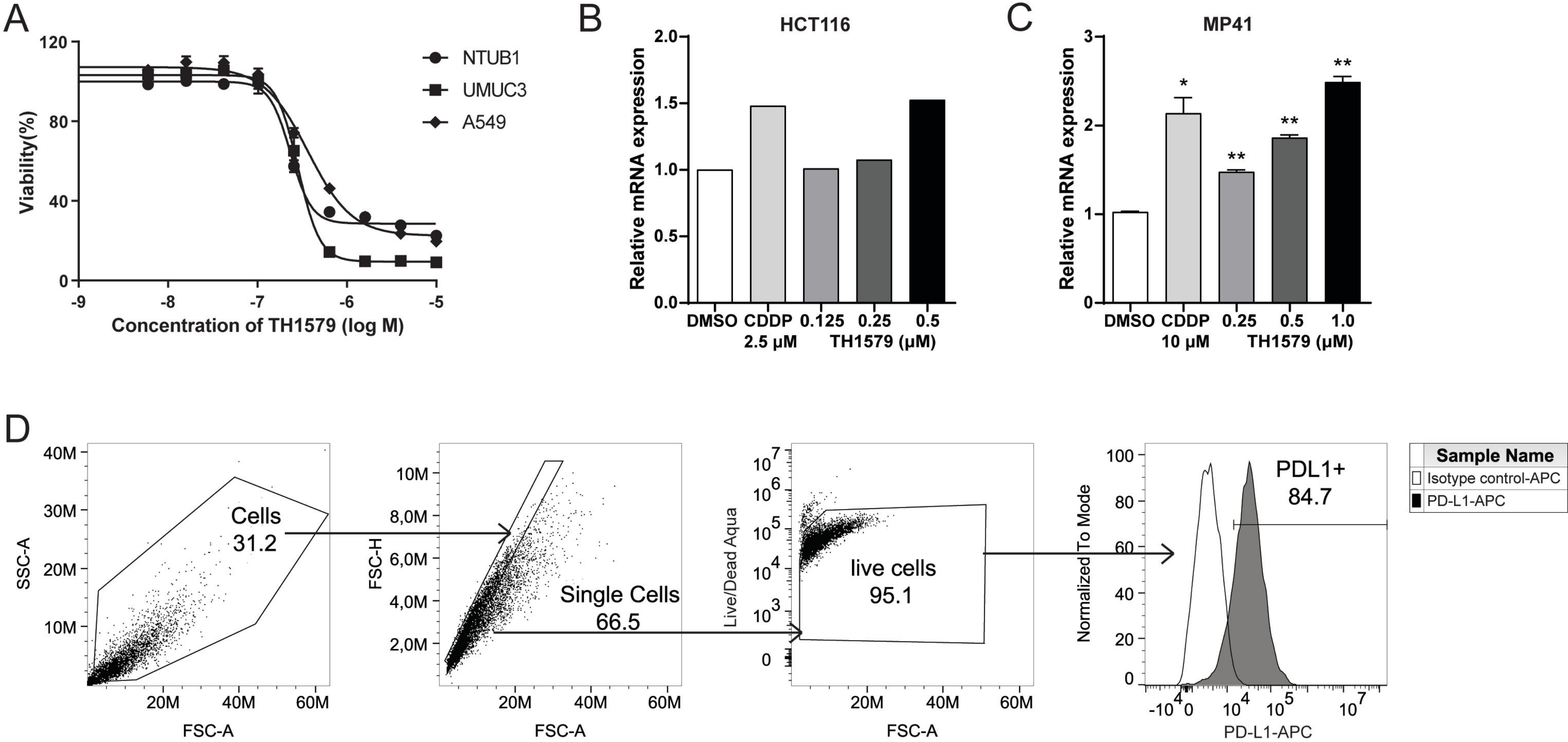
A
NTUB1
UMUC3
A549
Viability(%)
Concentration of TH1579 (log M)
B
HCT116
Relative mRNA expression
DMSO
CDDP 2.5 µM
0.125
0.25
0.5
TH1579 (µM)
C
MP41
Relative mRNA expression
DMSO
CDDP 10 µM
0.25
0.5
1.0
TH1579 (µM)
D
Cells 31.2
SSC-A
FSC-A
Single Cells 66.5
FSC-H
FSC-A
live cells 95.1
Live/Dead Aqua
FSC-A
PDL1+ 84.7
Normalized To Mode
PD-L1-APC
Sample Name
Isotype control-APC
PD-L1-APC

Figure S2

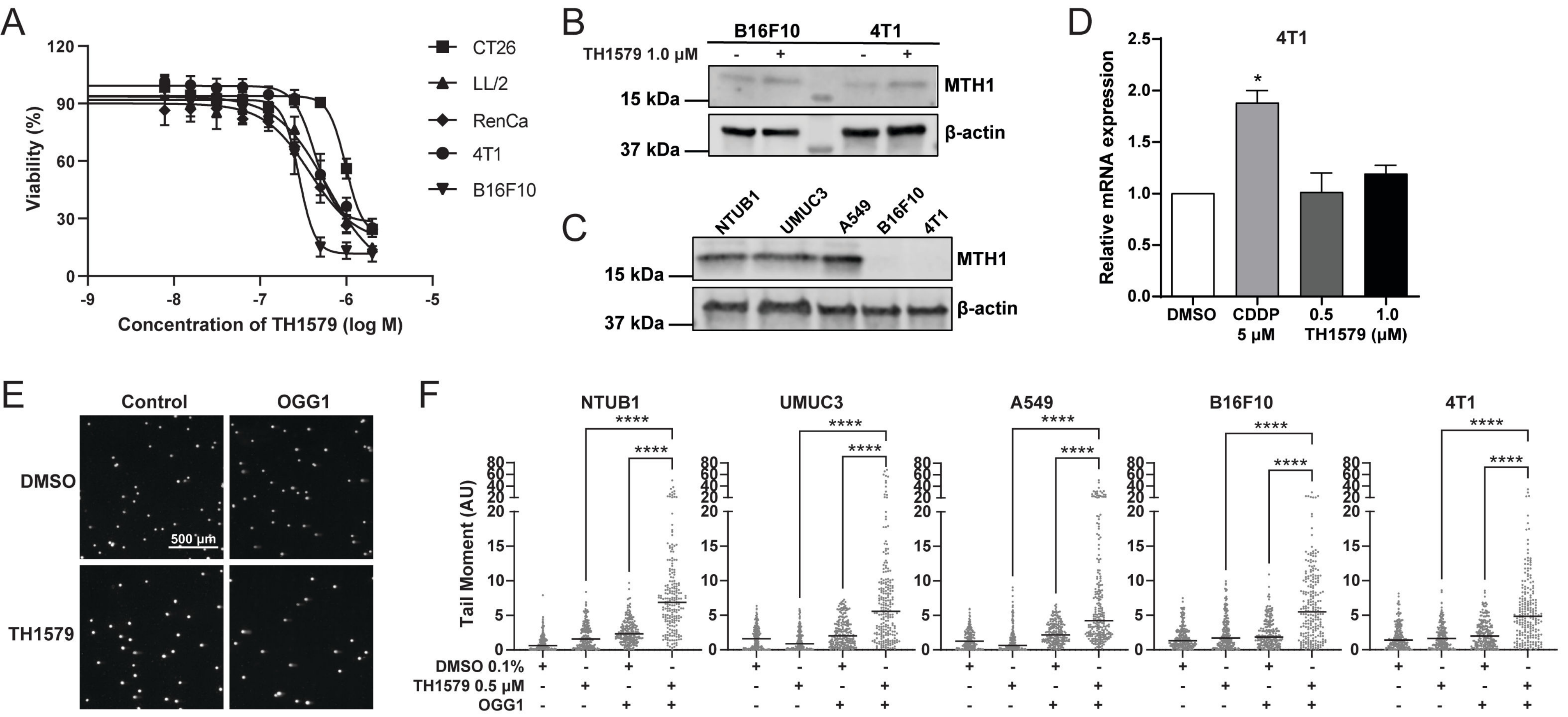
A
CT26
LL/2
RenCa
4T1
B16F10
Viability (%)
Concentration of TH1579 (log M)
B
B16F10
4T1
TH1579 1.0 μM
MTH1
β-actin
15 kDa
37 kDa
C
NTUB1
UMUC3
A549
B16F10
4T1
MTH1
β-actin
15 kDa
37 kDa
D
4T1
Relative mRNA expression
DMSO
CDDP 5 μM
0.5
1.0
TH1579 (μM)
E
Control
OGG1
DMSO
TH1579
500 μm
F
NTUB1
UMUC3
A549
B16F10
4T1
Tail Moment (AU)
DMSO 0.1%
TH1579 0.5 μM
OGG1

Figure S3

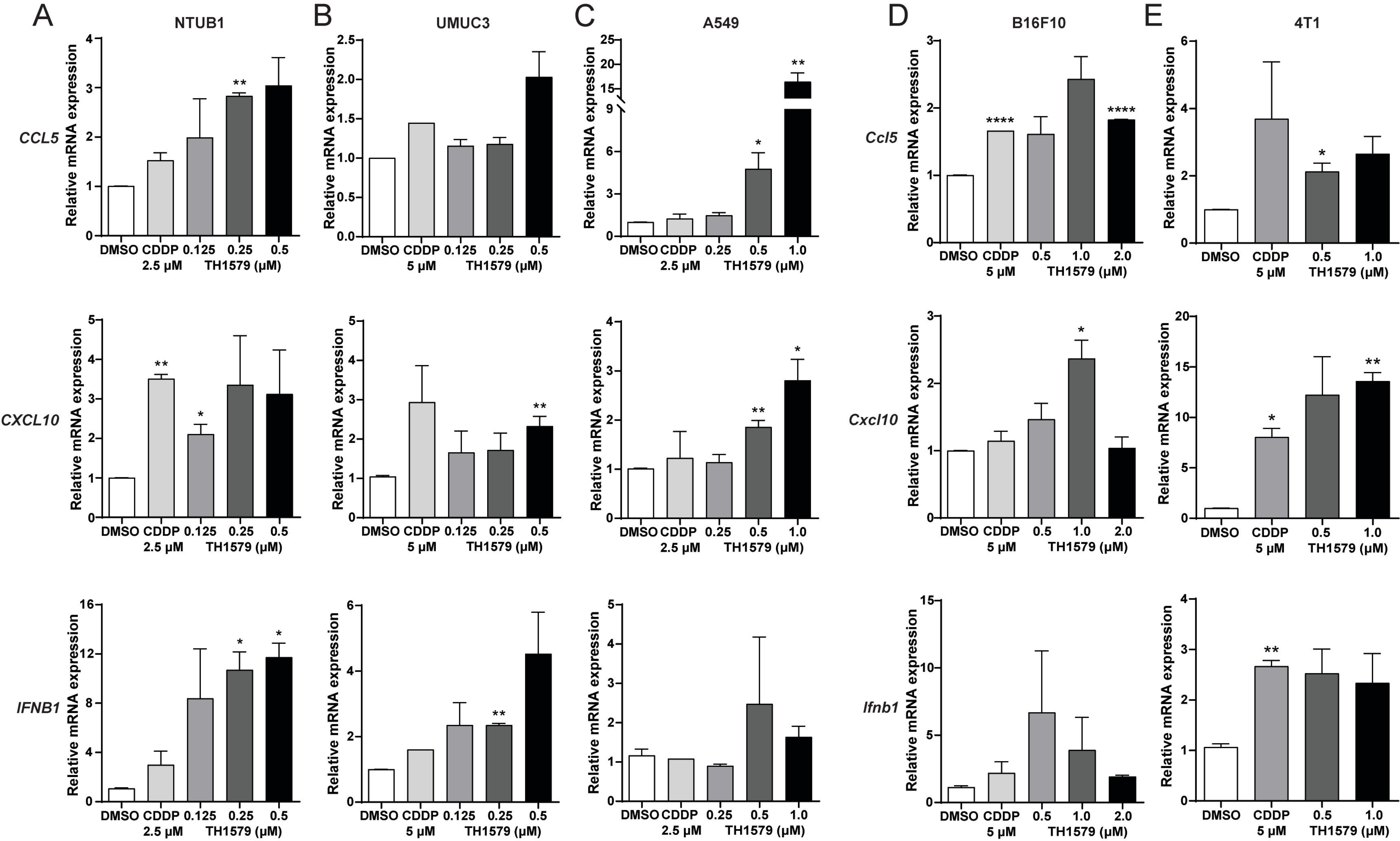
A
NTUB1
B
UMUC3
C
A549
D
B16F10
E
4T1
CCL5
CXCL10
IFNB1
Ccl5
Cxcl10
Ifnb1
Relative mRNA expression
DMSO
CDDP 2.5 μM
CDDP 5 μM
0.125
0.25
0.5
1.0
2.0
TH1579 (μM)
*
**
****

Figure S4

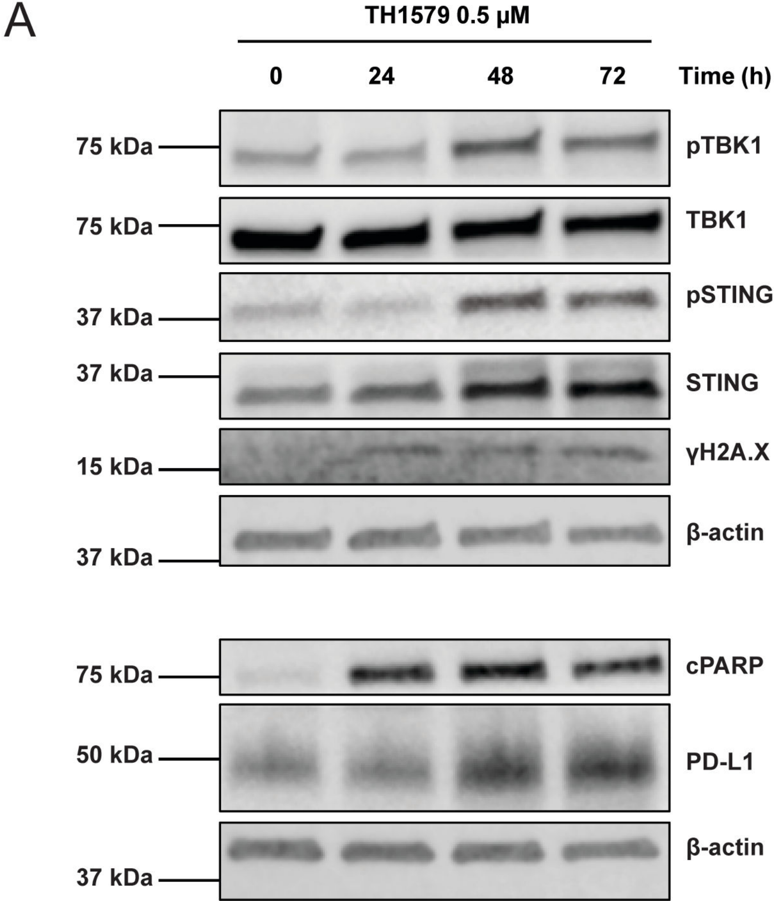
A
TH1579 0.5 µM
0
24
48
72
Time (h)
75 kDa
pTBK1
75 kDa
TBK1
pSTING
37 kDa
37 kDa
STING
γH2A.X
15 kDa
β-actin
37 kDa
cPARP
75 kDa
50 kDa
PD-L1
β-actin
37 kDa

Figure S5

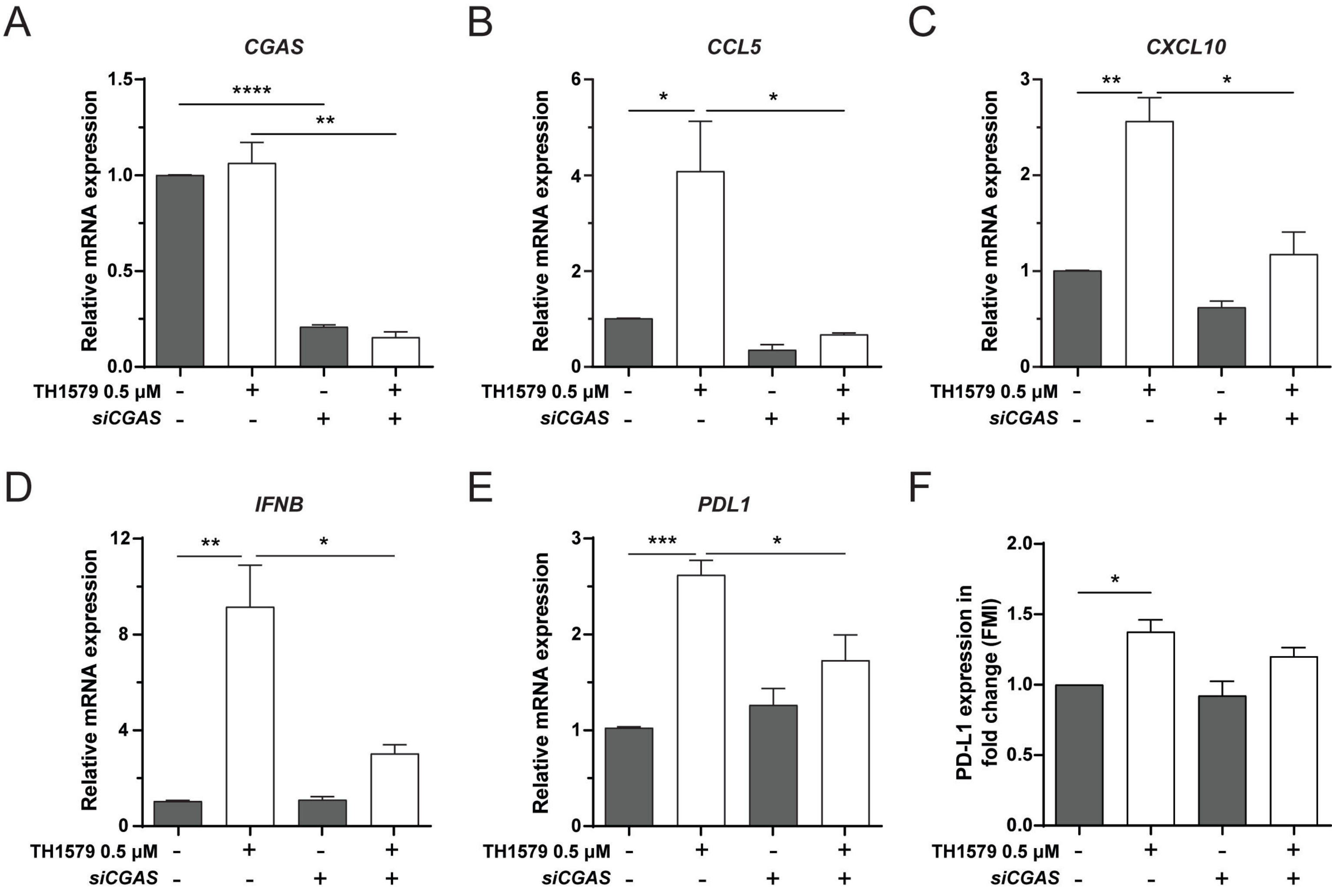
A
CGAS
Relative mRNA expression
1.5
1.0
0.5
0.0
****
**
TH1579 0.5 µM - + - +
siCGAS - - + +
B
CCL5
Relative mRNA expression
6
4
2
0
*
*
TH1579 0.5 µM - + - +
siCGAS - - + +
C
CXCL10
Relative mRNA expression
3
2
1
0
**
*
TH1579 0.5 µM - + - +
siCGAS - - + +
D
IFNB
Relative mRNA expression
12
8
4
0
**
*
TH1579 0.5 µM - + - +
siCGAS - - + +
E
PDL1
Relative mRNA expression
3
2
1
0
***
*
TH1579 0.5 µM - + - +
siCGAS - - + +
F
PD-L1 expression in fold change (FMI)
2.0
1.5
1.0
0.5
0.0
*
TH1579 0.5 µM - + - +
siCGAS - - + +

Figure S6

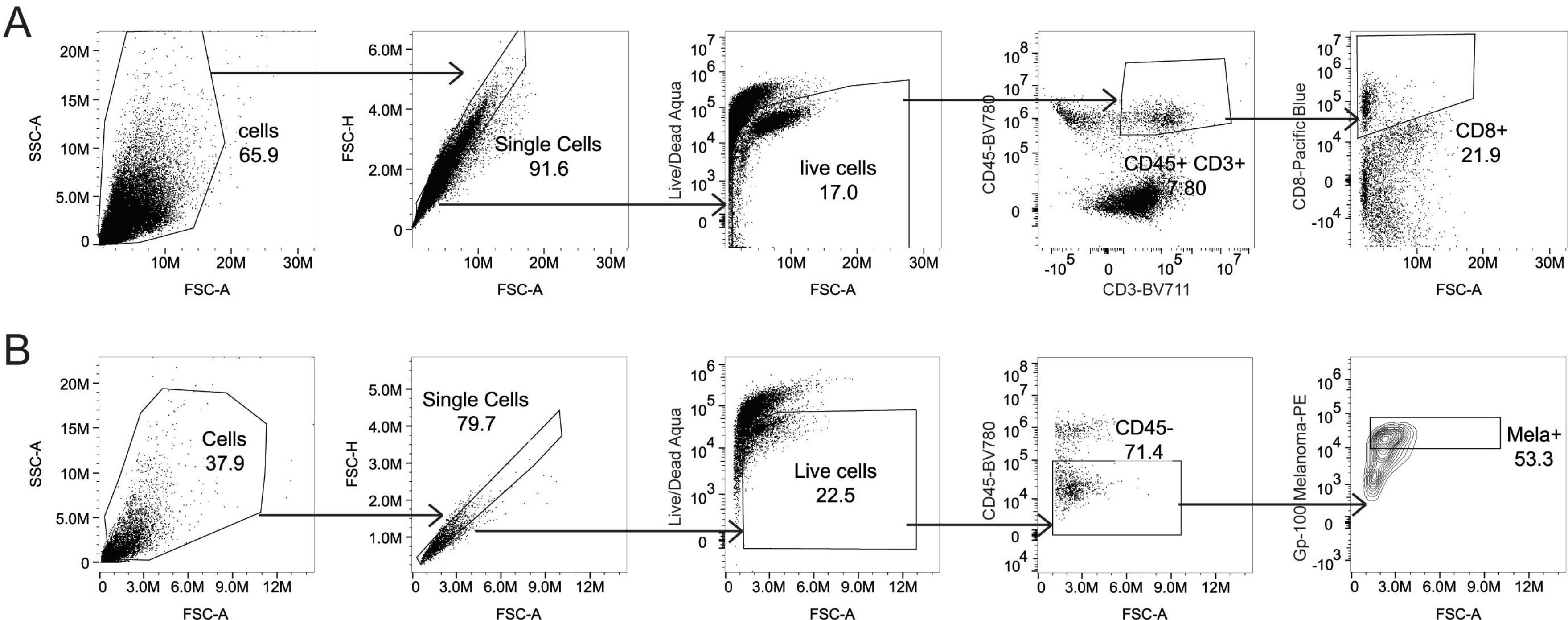

A
cells
65.9
Single Cells
91.6
live cells
17.0
CD45+ CD3+
7.80
CD8+
21.9
SSC-A
FSC-H
Live/Dead Aqua
CD45-BV780
CD8-Pacific Blue
FSC-A
CD3-BV711
B
Cells
37.9
Single Cells
79.7
Live cells
22.5
CD45-
71.4
Mela+
53.3
Gp-100 Melanoma-PE

Figure S7

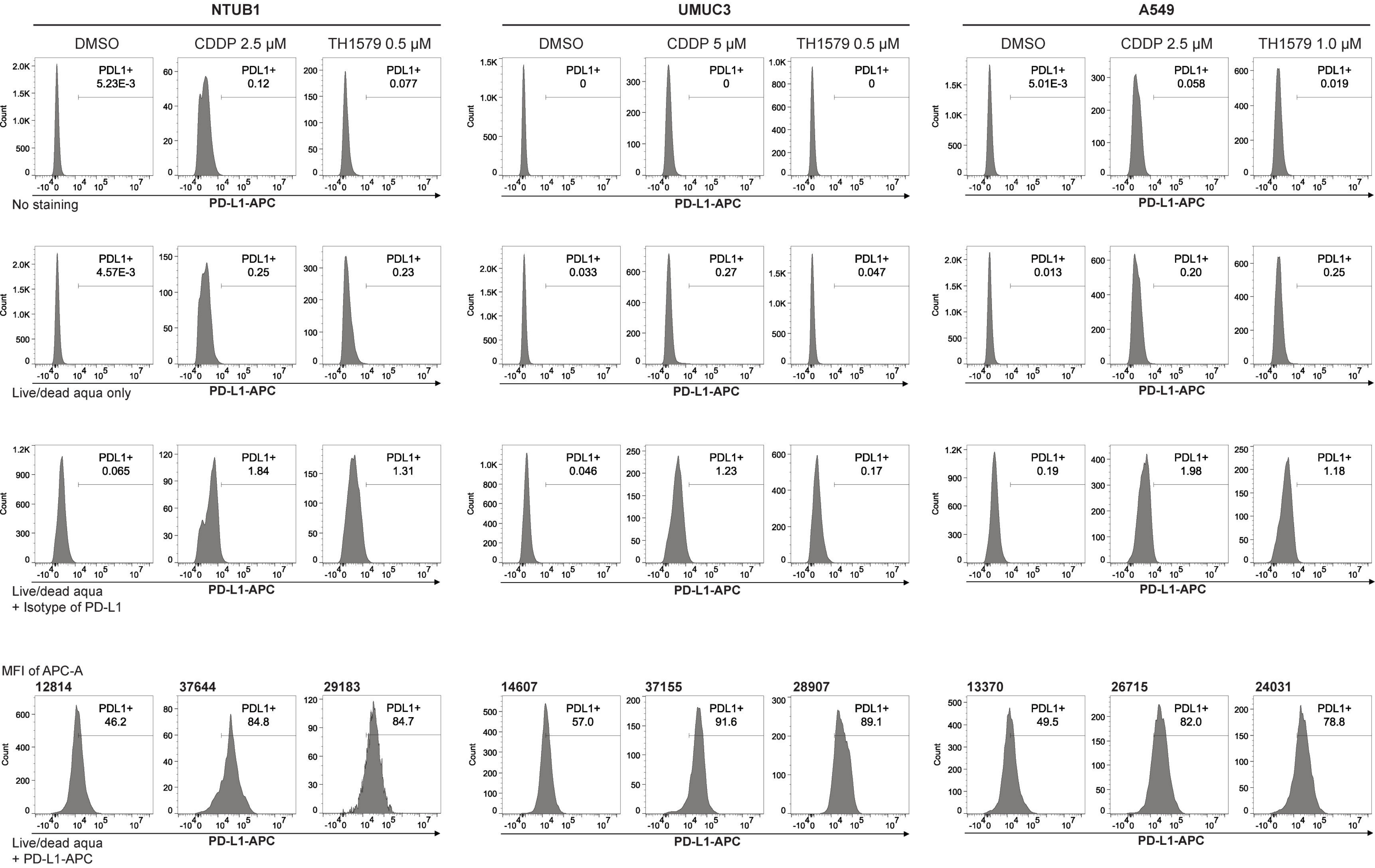

NTUB1
UMUC3
A549
DMSO
CDDP 2.5 μM
TH1579 0.5 μM
DMSO
CDDP 5 μM
TH1579 0.5 μM
DMSO
CDDP 2.5 μM
TH1579 1.0 μM
No staining
PDL1+ 5.23E-3
PDL1+ 0.12
PDL1+ 0.077
PDL1+ 0
PDL1+ 0
PDL1+ 0
PDL1+ 5.01E-3
PDL1+ 0.058
PDL1+ 0.019
Live/dead aqua only
PDL1+ 4.57E-3
PDL1+ 0.25
PDL1+ 0.23
PDL1+ 0.033
PDL1+ 0.27
PDL1+ 0.047
PDL1+ 0.013
PDL1+ 0.20
PDL1+ 0.25
Live/dead aqua + Isotype of PD-L1
PDL1+ 0.065
PDL1+ 1.84
PDL1+ 1.31
PDL1+ 0.046
PDL1+ 1.23
PDL1+ 0.17
PDL1+ 0.19
PDL1+ 1.98
PDL1+ 1.18
MFI of APC-A
12814
37644
29183
14607
37155
28907
13370
26715
24031
Live/dead aqua + PD-L1-APC
PDL1+ 46.2
PDL1+ 84.8
PDL1+ 84.7
PDL1+ 57.0
PDL1+ 91.6
PDL1+ 89.1
PDL1+ 49.5
PDL1+ 82.0
PDL1+ 78.8
Count
PD-L1-APC
